# 5 Years of Exercise Intervention Did Not Benefit Cognition Compared to the Physical Activity Guidelines in Older Adults, but Higher Cardiorespiratory Fitness Did. A Generation 100 Substudy

**DOI:** 10.3389/fnagi.2021.742587

**Published:** 2021-11-16

**Authors:** Daniel R. Sokołowski, Tor I. Hansen, Henning H. Rise, Line S. Reitlo, Ulrik Wisløff, Dorthe Stensvold, Asta K. Håberg

**Affiliations:** ^1^Department of Neuromedicine and Movement Science, Faculty of Medicine and Health Sciences, Norwegian University of Science and Technology, Trondheim, Norway; ^2^Department of Radiology and Nuclear Medicine, St. Olavs Hospital, Trondheim University Hospital, Trondheim, Norway; ^3^Cardiac Exercise Research Group, Department of Circulation and Medical Imaging, Faculty of Medicine and Health Sciences, Norwegian University of Science and Technology, Trondheim, Norway; ^4^School of Human Movement and Nutrition Science, University of Queensland, Brisbane, QLD, Australia

**Keywords:** neuropsychology, prevention, aerobic training, recall, executive abilities, seniors, cognitive aging, memory

## Abstract

**Background:** Aerobic exercise is proposed to attenuate cognitive decline in aging. We investigated the effect of different aerobic exercise interventions and cardiorespiratory fitness (CRF) upon cognition throughout a 5-year exercise intervention in older adults.

**Methods:** 106 older adults (52 women, age 70-77 years) were randomized into high-intensity interval training (HIIT; ∼90% peak heart rate), moderate-intensity continuous training (MICT; ∼70% peak heart rate), or control for 5 years. The HIIT and MICT groups performed supervised training twice weekly, while the control group was asked to follow the national physical activity guidelines (30 min of physical activity/day). At baseline, 1-, 3-, and 5-year follow-up, participants partook in cognitive testing (spatial memory, verbal memory, pattern separation, processing speed, working memory, and planning ability), underwent clinical testing, and filled out health-related questionnaires. Linear mixed models were used to assess the effects of the exercise group and CRF (measured as peak and max oxygen uptake) on each cognitive test. The effects of changes in CRF on changes in each cognitive test score throughout the intervention were also assessed. The associations between baseline CRF and cognitive abilities at the follow-ups were investigated using linear regressions.

**Results:** There was no group-by-time interaction on the cognitive measures, and neither HIIT nor MICT participation was associated with better cognitive performance than control at any time point during the 5-year intervention. All groups increased their CRF similarly during the 1st year and subsequently declined back to baseline levels after 5 years. A higher CRF was associated with higher processing speed throughout the intervention while increasing CRF during the intervention was associated with better working memory and worse pattern separation. Higher CRF at baseline predicted consistently better processing speed and verbal memory performance.

**Conclusion:** In this first 5-year randomized controlled trial investigating the effects of HIIT, MICT, and physical activity according to national guidelines on cognition, we observed no effect of exercise intervention group on cognition when compared to following the national physical activity guidelines. Still, the results showed that higher CRF and increasing CRF benefited multiple, but not all, cognitive abilities in older adults.

**Clinical Trial Registration:** www.ClinicalTrials.gov, identifier [NCT01666340].

## Introduction

With the world’s population getting older, successful aging, which biomedical theories define as minimizing physical and mental deterioration and disability (Bowling and Dieppe, 2005), is necessary for achieving sustainable global development (UN, 2017). Physical activity is suggested as an accessible and effective method for attenuating a cognitive decline in older adults and lowering the risk of dementia (Barnes and Yaffe, 2011; Sofi et al., 2011; Livingston et al., 2017; Zotcheva et al., 2018; Tari et al., 2019; Lin et al., 2020). The cardiovascular fitness hypothesis holds that cardiorespiratory fitness (CRF, also called “aerobic fitness” and “cardiovascular fitness”) is the physiological mediator of the beneficial effects of physical activity on cognition. Cardiorespiratory fitness refers to an individual’s maximal oxygen uptake that is defined as the maximal amount of oxygen delivered to working muscles during dynamic work with large muscle mass (VO_2__*max*_). The cardiovascular fitness hypothesis posits that greater CRF increases cerebral blood flow, which improves oxygen transport and metabolism in the brain, leading to improved neurotransmitter function, waste removal, and improved cognitive function (Voss, 2016). However, both the exact mechanisms through which physical activity and exercise benefit cognition and the efficacy of these mechanisms, remain debated (Di Liegro et al., 2019).

Observational studies show that physical activity in middle and old age reduces both age-related cognitive decline and dementia risk (Laurin et al., 2001; Andel et al., 2008; Ahlskog et al., 2011; Barnes and Yaffe, 2011; Gow et al., 2017; Livingston et al., 2017; Sabia et al., 2017; Hamer et al., 2018; Tari et al., 2019). Because observational studies usually focus on general physical activity, they cannot verify the cardiovascular fitness hypothesis. In contrast, exercise intervention studies have tested it (Colcombe and Kramer, 2003; Etnier et al., 2007; van Uffelen et al., 2008; Smith et al., 2010; Young et al., 2015). While some intervention studies reported substantial positive effects of exercise intervention across all cognitive domains (Colcombe and Kramer, 2003), other studies with similar interventions found modest or inconclusive effects (Smith et al., 2010; Young et al., 2015). Moreover, there is no consensus on optimal intensity (Smith et al., 2010; Gomes-Osman et al., 2018; Calverley et al., 2020), duration (Smith et al., 2010; Gomes-Osman et al., 2018), or type of exercise intervention (Colcombe and Kramer, 2003; Smith et al., 2010; Gomes-Osman et al., 2018; Northey et al., 2018; Cabral et al., 2019) for cognition.

As high-intensity interval training (HIIT) has greater positive effects on CRF than moderate-intensity continuous training (MICT, Calverley et al., 2020), the cardiovascular fitness hypothesis contends that HIIT provides greater cognitive benefits. HIIT is also shown to have the greatest positive effect on cardiovascular health at any age (Swain and Franklin, 2006; Calverley et al., 2020), an effect that might translate into better brain health. While many observational studies on physical activity last for decades and focus on long-term cognitive decline in older adults, intervention studies typically last from several weeks to 2 years (Ellis et al., 2009; Ngandu et al., 2015). Consequently, intervention studies have been unable to uncover more long-term effects of aerobic exercise training on cognition.

The purpose of this study was to assess the validity of the cardiovascular fitness hypothesis on cognition in healthy older adults. We examined both the effects of supervised aerobic exercise training at different intensities and the effects of CRF *per se* and change in CRF throughout a 5-year intervention period on cognition. We used a randomized controlled trial (RCT) design, with participants assigned to supervised training twice a week with HIIT or MICT intervention, or a control condition which was asked to follow the government guidelines of 30 min physical activity per day. The study lasted 5 years. We conducted assessments, including cognitive testing and CRF measured as VO_2__*peak*_, at baseline, 1, 3, and 5 years.

According to the cardiovascular fitness hypothesis, we predicted that the HIIT intervention would have the largest positive effect on cognition by slowing age-related cognitive decline across all domains. We hypothesized that high CRF was associated with better cognitive performance throughout the intervention. Processing speed, as well as pattern separation, and executive functions (represented in this study by planning ability and working memory) should be linked to CRF (Colcombe and Kramer, 2003; Angevaren et al., 2008; Smith et al., 2010; Bolz et al., 2015; Gomes-Osman et al., 2018; Bernstein and McNally, 2019; Cabral et al., 2019). We also hypothesized that increasing CRF during the intervention would be associated with positive changes in the same cognitive domains at each timepoint. Finally, we examined if baseline CRF predicted better cognitive abilities at later time points to investigate the presence of the long-term effect of CRF since the duration of the positive effect of CRF on cognition remains unknown. The study was part of the Generation 100 Study, a 5-year RCT (Stensvold et al., 2015), which reported a trend toward reduced mortality and general health benefits in the HIIT compared to the other groups (Stensvold et al., 2020).

## Materials and Methods

### Study Population

The participants in the Generation 100 RCT (NCT01666340, ClinicalTrials.gov registry, Stensvold et al., 2015) are inhabitants of Trondheim County, Norway, registered in the National Population Registry. The project was approved by the Regional Committee for Medical Research Ethics (REC South East B; REK 2012/381 B). All participants signed an informed written consent form before joining the study.

All inhabitants born between 1936 and 1942 received an invitation letter (*n* = 6966). Altogether, 1790 declared interest while 1422 declined. Of 1790 interested, 223 withdrew before or during the baseline examination or were excluded. In total, 1567 (777 men, 790 women) were included in the study. The criterion for inclusion was having the physical ability to take part in an exercise intervention. There were three criteria for exclusion: somatic diseases that precluded exercise, dementia, or participation in other exercise trials. 49 people were excluded.

Before randomization in the Generation 100 study, the participants were asked if they were interested in also taking part in cognitive testing during the 5-year intervention period. The participants were stratified by sex and cohabitation status before being randomized 2:1:1 into control (*n* = 780), HIIT (*n* = 400), and MICT (v387) groups.

Baseline data collection started in August 2012 and lasted till June 2013. Follow-ups were performed 1, 3, and 5 years after baseline data collection with 5-year data collected between August 2017 and June 2018.

### Study Sample

Out of the 1567 participants in the Generation 100 Study, 111 (55 men, 56 women) were initially interested in also performing cognitive tests. Of these, four withdrew before testing and one was excluded due to a preexisting neurosurgical condition, leaving 106 participants (54 men, 52 women). The substudy was approved by the Regional Committee for Medical Research Ethics, Central Norway (2012/849).

#### Intervention

The control group followed the Norwegian Health Authorities’ physical activity recommendations of 30 min of moderate-intensity physical activity almost every day. The supervised exercised groups were assigned to 2 weekly sessions of either HIIT consisting of 10-min warm-up with subsequent 4 × 4-min intervals at 85-95% of peak heart rate, corresponding to about Borg 16-20 on the rating of perceived exertion scale (Borg, 1982), or MICT consisting of 50 min of continuous training at 70% of peak heart rate, corresponding to approximately 13 on the scale (Stensvold et al., 2015). All HIIT and MICT participants had to join a mandatory spinning class every 6th week, where they exercised with a heart rate monitor to ascertain compliance with the prescribed training intensity. The study protocol was described in detail elsewhere (Stensvold et al., 2015).

Adherence to the exercise intervention or physical activity guidelines, the weekly frequency, duration, and intensity of exercise sessions in control, MICT, and HIIT, as well as the frequency of performing different types of activities were based on self-report questionnaires (Aspenes et al., 2011) filled in at the time of the clinical examination at 1, 3, and 5 years. Adherence for each group was calculated as the number of participants adhering to the prescribed exercise program divided by the total number of participants in the group at that time point and presented as a percentage. For HIIT, adherence was defined as exercising at least 30 min at 15 on the Borg scale per week. For MICT, adherence was defined as at least 30 min at 11-14 on the Borg scale per week. For the controls, adherence was defined as at least 75 min of physical activity per week, intensity was not considered for this group (Stensvold et al., 2020). The frequency of performing different types of activities was based on a question: “How often do you do the following?: (1) Walking: (a) as a way of transport, (b) recreational walking, (c) hiking in nature; (2) Cycling; (3) Swimming; (4) Skiing (in winter); (5) Using fitness center; (6) Organized sports; (7) Other activities”. The response options were: “Never” scored as 0; “Rarely” scored as 0.25; “1−3 times a month” scored as 0.5; “once a week” scored as 1, “2−3 times a week” scored as 2.5; “4−6 times a week” scored as 5; and “Daily” scored as 7. We reported weekly frequencies based on the scores.

#### Clinical and Physical Data

Participants filled in standardized questionnaires to obtain data on sex, date of birth, level of education, cohabitation status, smoking habits, and several health measures (Stensvold et al., 2015). Psychological health was assessed with a validated, Norwegian version of the Hospital Anxiety and Depression Scale (HADS) questionnaire (Zigmond and Snaith, 1983; Mykletun et al., 2001) with a total score reported. We created an index for sleep problems based on three questions: “How often in the last 3 months have you: (1) Had difficulty with falling asleep at night?; (2) Woken up repeatedly during the night?; and (3) Woken too early and couldn’t get back to sleep?” The response options were: “Never/seldom” scored as 1; “Sometimes” scored as 2; and “Several times a week” scored as 3 (Bragantini et al., 2019). We reported the total score. We used the Norwegian validated version of the Montreal Cognitive Assessment (MoCA) at the end of the intervention to assess the development of possible mild cognitive impairment or dementia while in the study (Nasreddine et al., 2005). The total score was reported.

We employed standard practices to acquire clinical measurements (e.g., blood pressure, heart rate, body composition) and fasting blood samples (i.e., glucose, lipids) analysis (Stensvold et al., 2017). Testing of oxygen uptake was performed on a motor-driven treadmill (Woodway USA Inc., PPS 55, Waukesha, WI, United States) or a stationary bike (Lode B.V., Zernikepark 16, 9747AN, Groningen, Netherlands) at the NextMove Core Facilities, St. Olavs Hospital, Norway. We tested participants with previous cardiovascular diseases under ECG monitoring and followed the guidelines for exercise testing by the American College of Cardiology/American Heart Association (Gibbons et al., 1997). Stationary bikes were only used for participants who were unable to walk on a treadmill (i.e., unstable or short-term injury). Participants that were tested on a bike had their follow-up tests using the same ergometer cycle throughout the study (Cortex MetaMax II, Leipzig, Germany). The VO_2_ testing and calibration procedure have been described previously (Stensvold et al., 2017). In short, a facemask (Hans Rudolph, United States) connected to the gas-analyzer was attached to the participants before initiating the test and used throughout. After a 10-min warm-up at moderate intensity, a protocol was used where the load was increased each minute and a half by 1 km/h or 2% inclination. The main criterion for terminating the test was voluntarily exhaustion (i.e., VO_2__*peak*_) or reaching a plateau in oxygen uptake despite the increased load (i.e., VO_2__*max*_), which was defined as no more than 2 mL⋅min^–1^⋅kg^–1^ between two 30-s segments. The helping criteria were reaching Borg scale ≥ 17 and RER ≥ 1.0 for VO_2__*peak*_ and RER ≥ 1.05 for VO_2__*max*_. To calculate VO_2__*peak*_, the mean of the three successively highest 10-s VO_2_ registrations was used. As not all measurements met the VO_2__*max*_ criteria, VO_2__*peak*_ and VO_2__*max*_ measures were combined and referred to as VO_2__*peak*_ in the main analyses. Supplemental analyses were performed in participants who reached VO_2__*max*_, defined as reaching a plateau in oxygen uptake together with RER ≥ 1.05, with the measurement done on a treadmill since VO_2__*max*_ measures obtained on exercise bike tend to be lower than those obtained on a treadmill (Beltz et al., 2016). Peak heart rate (HR_*peak*_) was determined by the five beats above the highest observed heart rate (Polar Electro Oy, Finland) during the ergospirometry test, and used to determine exercise intensity.

#### Cognitive Testing

We collected the cognitive data in conjunction with the clinical and physical testing. The participants performed cognitive tests using Memoro (memoro.no), a validated, self-administered, web-based neuropsychological test platform for Norwegian-speaking participants (Hansen et al., 2015, 2016), proprietary to Tor Ivar Hansen and Asta Kristine Håberg. The Memoro tests were performed in a fixed order to allow for the delayed recall of the verbal memory test and for verbal and spatial tasks not to interfere. The test order was verbal memory, planning ability, processing speed, verbal memory delayed recall, pattern separation, spatial memory, working memory. Two variants of the battery were administered to all the participants alternately, set one at baseline and 3 years, set two at 1 year and 5 years.

##### Spatial Memory

The Objects in Grid Test, adapted from the Location Learning Test (Bucks and Willison, 1997), assesses immediate recall of objects’ locations. Participants had 90 s to memorize the locations of 18 colored drawings placed on a 6 × 6 grid. After the encoding period, all objects were moved outside the grid and participants had to drag and drop each object into its original location. All objects had to be positioned into the grid to proceed. Their scores were based on the number of objects they placed correctly.

##### Verbal Memory

The Verbal Memory Test, analogous to The California Verbal Learning Test Version 2 (Woods et al., 2006), is a traditional auditory word list test where participants memorize a target list of 16 words that are presented four times. In each trial, words were presented with a 3-s interval between the audio clips, for a total of about 55 s. After each presentation, participants typed in the words they recalled. Subsequently, a distraction list containing 16 words was presented and immediately recalled. Then the target list was recalled again. After approximately 15 min with other non-verbal tests, participants performed a delayed recall of the target list. The Verbal Memory score is the sum of correctly recalled words after each of the learning trials, together with the immediate recall trial, as well as the number of correctly recalled words in the delayed trial.

##### Pattern Separation

We assessed the pattern separation aspect of episodic memory by using an adaptation of the Behavioral Pattern Separation (BPS) Task – Object Version (BPS-O, Stark et al., 2013). Participants saw 108 successive images and had to indicate whether the image currently being presented was novel, i.e., presented for the first time, identical, or similar to a previously presented image. The stimuli were presented until the response was registered. The BPS score was calculated as the ratio of correctly identified similar items, minus the ratio of similar responses given to items not previously seen (Stark et al., 2013).

##### Processing Speed

The Processing Speed test was based on the Number Comparison and the Letter Comparison Tests (Salthouse and Babcock, 1991). It provides a measure of perceptual speed involving simple same/different decisions. Participants judged if pairs of geometrical shapes or numbers were identical or different. They responded by hitting the “F” (different) or “L” (identical) key as quickly as possible without making mistakes. The test consisted of six blocks of increasingly complex stimuli, each lasting 30 s. The total score is the number of correct trials.

##### Working Memory

We assessed working memory by using the Digit Span Backwards test (DSB, Wechsler, 2003). Participants memorized digits presented on the screen and typed them in a reversed order afterward. Each stimulus appeared on the screen for 2 s. The difficulty level increased progressively after each time participants successfully remembered a sequence, starting with two digits in the first trial and ending with up to ten digits. When a participant made an error, the following trial did not increase in difficulty. The test finished after 18 trials or was discontinued after three consecutive errors. We calculated the score by counting the maximal digit span.

##### Planning Ability

We evaluated the planning ability aspect of executive functioning with a modified version of the Tower of London test (Shallice et al., 1982). Participants were instructed to recreate a pattern of discs on pins by moving the discs one at a time, using as few moves as possible, while obeying certain rules. The test consisted of 15 trials which became progressively more difficult every third trial, requiring from one to five moves to solve. There were no time constraints. Points were given only for solving trials with the minimum number of moves, and more difficult trials were awarded more points. We calculated a composite score by adding the acquired points (Rainville et al., 2002).

### Statistical Analysis

#### Sample Size

The sample size was calculated based on a lower incidence of decline in performance on cognitive tests across the 5 years in the supervised training groups compared to the control group in which performance was expected to decline between 33 and 54% (Dodge et al., 2006; Howieson et al., 2008; Terrera et al., 2010; Yaffe et al., 2010) leading to group sizes between 9 and 19 participants required to find differences with an alpha of 0.05 and power of 80%.

#### Demographics, Physical Measures, and Clinical Variables

Demographics, physical measures, and clinical variables at every time point were compared between the control-, MICT-, and HIIT- groups with ANOVA, Kruskal – Wallis test, or Pearson Chi-Square as appropriate.

#### Adherence and Types of Performed Activities

Group differences in adherence to the appointed HIIT, MICT, or control protocol were assessed with Pearson Chi-Square or Fischer exact at each time point. Group differences in the number of exercise sessions per week, the duration of those sessions, the resulting exercise duration per week, and the exercise intensity, as well as in the frequency of performing various types of activities, were assessed with Kruskal – Wallis tests and Dunn’s tests.

#### Cardiorespiratory Fitness Throughout the Intervention

Changes in CRF (i.e., VO_2__*peak*_) in each group throughout the intervention, as well as the potential effect of whether the measurement was classified as VO_2__*peak*_ or VO_2__*max*_, were assessed with a linear mixed model (LMM) with CRF as the outcome variable, and time, time^∗^group interaction, and whether the measurement was classified as VO_2__*peak*_ or VO_2__*max*_, as predictive variables. All the predictive variables were coded as dummy variables with baseline, the control group, and VO_2__*max*_ as references, controlling for age, sex, and education. The same method was performed in the supplementary analysis that included only the participants who reached VO_2__*max*_ on a treadmill.

#### Group and Cardiorespiratory Fitness Effects on Cognitive Scores

Baseline differences in cognitive scores between the participants who remained in the study throughout its duration and those who withdrew were analyzed with the Mann-Whitney *U*-test using the raw scores.

For the linear mixed models and the linear regressions, standard scores (z-scores) of each cognitive test were used. Standard scores were calculated for all time points together in LMMs and for each time point separately in linear regressions. This was done to make the interpretation of coefficient estimates easier and the effect sizes comparable between the different cognitive outcome variables while keeping the results identical to those we would get by working with the raw values. For example, a coefficient estimate of 0.1 translates into a difference of 0.1 standard deviations (SD) above the mean (compared to the reference group, i.e., control group) in case of categorical predictive variables or to an increase of the outcome variable of 0.1 SD for each one-unit-increase of the predictive variable in case of continuous predictors.

First, we assessed the effect of participating in the HIIT and MICT groups on cognitive abilities during the 5-year intervention using LMM in which CRF at each timepoint was included (Model 1). For each cognitive test, we entered the standardized score as the outcome variable, with time, group^∗^time interaction, and CRF over time as predictive variables, and controlled for age at inclusion, sex, and education. We entered subjects as the random effect term and treated time and group^∗^time interactions as dummy variables with baseline cognitive score and the control group as references as described by Twisk et al. (2018).

The effect of change in CRF on change in cognitive performance during the intervention was assessed in an LMM (Model 2). Model 2 was similar to Model 1, but in order to focus on the effect of changes in CRF on cognition throughout the intervention, baseline CRF was subtracted from the CRF value at baseline, 1, 3, and 5 years, thus giving the value of 0 for baseline and the change in CRF between baseline and each time point for later time points.

Finally, to assess if baseline CRF predicted cognition at later time points, linear regression models were performed (Model 3). For each analysis in Model 3, we entered the standardized cognitive score (at 1, 3, and 5 years, separately) as the outcome variable, CRF at baseline as a predictive variable, and controlled for age at the time of testing, sex, and education.

As described above, CRF in Models 1-3 was based on both VO_2__*max and*_ VO_2__*peak*_ measurements. The replication of these models including only the participants who achieved VO_2__*max*_ on a treadmill, with RER ≥ 1.05, can be found in the Supplementary.

The statistical analyses were performed in IBM SPSS Statistics for Windows (version 27) and Stata (version 17.0). The clinical and physical data is reported as a mean with a standard deviation. For categorical variables, numerical amounts are reported. For linear mixed models and linear regression analysis, coefficients and confidence intervals are reported.

*p* < 0.05 were considered statistically significant. Our interpretation of the results is based on the uncorrected *p*-Values, as per protocol. For completeness, corrections for multiple comparisons were performed using the Holm-Bonferroni method. As multiple *p*-Values were obtained in each linear mixed model, corrections were applied separately to each cognitive test score. In the case of linear regression, the correction was applied across the three time points. Corrections were applied jointly for main and supplementary analyses. Results that remained statistically significant after the corrections were marked with “#” in the tables. Please consider this when interpreting the statistical results.

## Results

**FIGURE 1:**
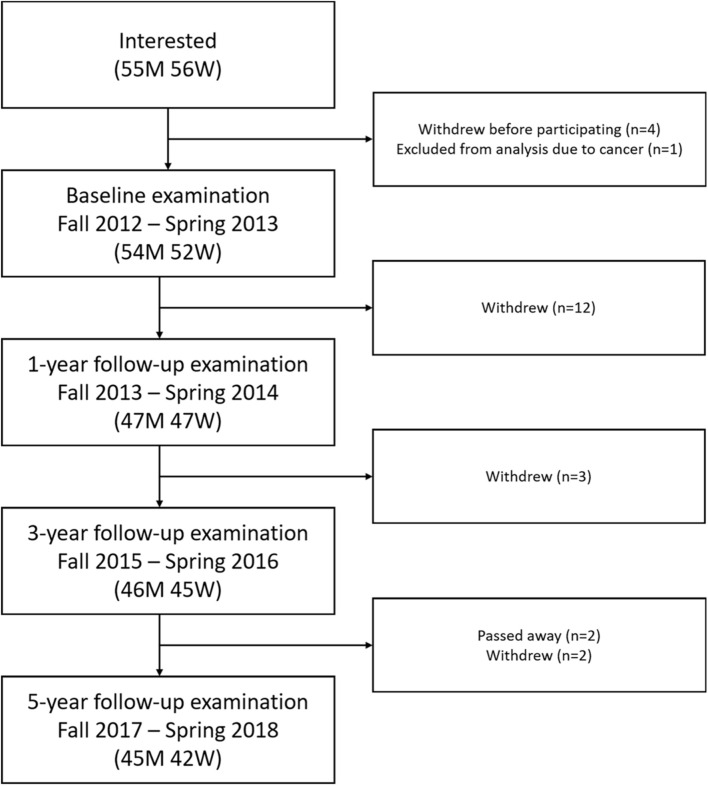
Flowchart of inclusion and attrition. M: men; W: women.

Overall, 106 participants (54 men, 52 women) were included at baseline and 87 participants (45 men, 42 women) remained in the study at 5-year follow-up (Figure 1). Participants who withdrew were similar in sex, education, and most cognitive scores as those who remained, but they were older and had lower processing speed at baseline (Table 1). The number of participants who reached VO_2__*max*_ on a treadmill was 65 (33 men, 32 women) at baseline and 39 (18 men, 21 women) at the 5-year follow-up.

**TABLE 1:** Baseline differences between participants who remained in the study for the entire duration and those who withdrew from the study.

	Not withdrawn	Withdrawn	*p*-Value
Age (years, mean [SD])^*a*^	72.4 [1.9]	73.4 [1.7]	0.01*
Number of participants (M/W)^*b*^	45/42	9/10	0.73
Education (P/S/T)^*b*^	7/19/60	2/9/8	0.06
CRF (mean [SD])^*a*^	30.4 [6.4]	28.7 [6.4]	0.35
Spatial memory (mean [SD])^*a*^	7.5 [3.7]	6.0 [4.0]	0.10
Verbal memory (mean [SD])^*a*^	71.2 [12.2]	66.4 [15.3]	0.26
Pattern separation (mean [SD])^*a*^	0.22 [0.17]	0.22 [0.20]	0.97
Processing speed (mean [SD])^*a*^	44.4 [11.8]	35.5 [9.2]	0.003*
Working memory (mean [SD])^*a*^	5.3 [1.6]	5.5 [1.6]	0.94
Planning ability (mean [SD])^*a*^	29.3 [8.2]	26.6 [9.6]	0.42

*p < 0.050, ^a^Kruskal – Wallis test, ^b^Pearson Chi-Square. M: men; W: women; P: primary education; S: secondary education; T: tertiary education; CRF: cardiorespiratory fitness measured as VO_2peak_.

### Demographic, Clinical, and Physical Data

Table 2 shows the demographic, clinical, and physical characteristics of participants in the MICT, HIIT, and control groups at baseline and 5 years. Supplementary Table 1 shows the same characteristics for the 1-year and 3-year follow-ups. Most participants had completed higher education and had clinical measurements within normal ranges. Those characteristics were retained in those who remained in the study and none of the participants developed MCI during the 5-year follow-up. We found no significant group differences concerning the demographic, clinical, or physical variables at any time point (Table 2 and Supplementary Table 1), other than total cholesterol, which, compared to the control group, was lower in MICT at 3 years (*z* = −2.71, *p* = 0.003). Note that all groups’ total cholesterol values were in the normal range.

**TABLE 2:** Demographics, physical measures, and clinical variables for the control group, the moderate intensity continuous training group (MICT), and the high intensity interval training group (HIIT) at baseline and 5-year follow-up.

	Baseline				5-year follow-up			
	Control	MICT	HIIT	*p*-Value	Control	MICT	HIIT	*p*-Value
Sex (M/W)^1^	23/25	11/13	20/14	0.53^*d*^	19/18	10/11	16/13	0.87^*d*^
Age (years)^2^	72.4 [1.9]	72.2 [1.8]	72.7 [2.2]	0.78^*a*^	77.4 [1.8]	77.6 [1.7]	77.7 [2.1]	0.78^*a*^
Education (P/S/T)^1^	4/16/28	3/5/16	2/7/24	0.59^*d*^	2/11/24	3/3/15	2/5/21	0.50^*d*^
Living alone (Y/N)^1^	15/33	7/16	9/23	0.96^*d*^	12/22	4/15	7/19	0.53^*d*^
Current smoker (Y/N)^1^	5/43	1/22	3/30	0.69^*d*^	4/28	1/18	2/24	0.68^*d*^
Height (cm)^2^	169.0 [9.7]	171.6 [7.5]	171.0 [8.6]	0.13^*b*^	168.7 [10.3]	170.0 [8.2]	168.6 [7.9]	0.24^*b*^
Weight (kg)^2^	74.1 [13.2]	75.7 [9.9]	76.8 [13.4]	0.76^*b*^	74.0 [14.8]	75.1 [10.8]	74.1 [11.5]	0.69^*b*^
Waist circumference (cm)^2^	93.3 [11.0]	93.5 [9.2]	94.5 [11.1]	0.99^*b*^	94.5 [12.8]	94.8 [8.9]	94.7 [11.1]	0.94^*b*^
Muscle mass (%)^2^	28.3 [6.1]	29.4 [4.4]	30.1 [5.5]	0.25^*b*^	27.8 [5.7]	27.9 [5.0]	28.0 [4.8]	0.35^*b*^
Fat (%)^2^	30.3 [8.0]	29.5 [7.8]	28.1 [7.0]	0.67^*b*^	30.6 [8.2]	31.4 [7.5]	30.0 [5.8]	0.86^*b*^
BMI (kg/m^2^)^2^	25.9 [3.3]	25.9 [3.5]	26.1 [3.3]	0.97^*b*^	25.9 [3.8]	26.0 [3.6]	26.0 [2.5]	0.97^*b*^
RHR (beats/min)^2^	63.4 [9.0]	65.0 [8.9]	63.2 [10.4]	0.79^*b*^	61.2 [8.6]	62.3 [6.2]	60.4 [8.3]	0.78^*b*^
DBP right (mmHg)^2^	74.2 [8.2]	77.4 [8.7]	75.8 [8.7]	0.33^*b*^	77.0 [7.2]	76.7 [13.0]	75.4 [11.5]	0.83^*b*^
SBP right (mmHg)^2^	135.4 [17.5]	132.3 [14.0]	133.6 [18.9]	0.78^*b*^	135.5 [17.5]	132.6 [18.7]	136.4 [19.1]	0.77^*b*^
Total cholesterol (mmol/L)^2^	5.9 [1.0]	5.5 [0.6]	5.7 [1.1]	0.09^*a*^	5.6 [1.1]	4.9 [1.0]	5.6 [0.9]	0.10^*a*^
HDL (mmol/L)^2^	1.9 [0.6]	1.8 [0.5]	1.9 [0.7]	0.89^*a*^	1.71 [0.5]	1.7 [0.5]	1.8 [0.5]	0.62^*a*^
LDL (mmol/L)^2^	3.6 [1.0]	3.2 [0.7]	3.3 [1.0]	0.22^*a*^	3.3 [1.0]	2.8 [1.0]	3.2 [0.8]	0.21^*a*^
Glucose (mmol/L)^2^	5.6 [0.6]	5.4 [0.7]	5.6 [0.8]	0.12^*a*^	5.3 [0.4]	5.4 [1.1]	5.4 [0.6]	0.55^*a*^
HbA1c (%)^2^	5.6 [0.3]	5.6 [0.3]	5.6 [0.5]	0.74^*a*^	5.3 [0.3]	5.5 [0.5]	5.4 [0.5]	0.67^*a*^
hsCRP (mg/L)^2^	1.5 [1.1]	2.1 [3.8]	3.0 [4.9]	0.43^*a*^	3.8 [5.7]	2.2 [3.7]	2.8 [4.9]	0.18^*a*^
TG (mmol/L)^2^	1.0 [0.4]	1.0 [0.4]	1.1 [0.6]	0.89^*a*^	1.0 [0.4]	0.9 [0.4]	1.0 [0.4]	0.18^*a*^
HADS total score^2^	6.3 [4.2]	6.0 [4.0]	5.4 [3.2]	0.64^*a*^	7.6 [4.7]	7.0 [5.5]	5.7 [3.6]	0.26^*a*^
Sleep problem index^2^	5.1 [1.5]	5.6 [1.7]	4.9 [1.5]	0.36^*a*^	5.4 [1.5]	5.6 [1.9]	5.1 [1.3]	0.72^*a*^
MoCA score^*e*, 2^	−	−	−	−	25.7 [2.7]	26.6 [3.3]	26.4 [2.8]	0.27^*a*^

**Cardiorespiratory fitness testing**								

CRF (mL⋅kg^–1^⋅min^–1^)^2^	30.3 [6.6]	29.8 [5.8]	30.1 [6.8]	0.98^*b*^	30.1 [7.6]	28.6 [5.3]	30.5 [6.1]	0.68^*b,c*^
VO_2__*max*_ or VO_2__*peak*_^*f*, 1^	31/17	11/11	21/12	0.29^*d*^	19/12	7/11	13/11	0.41^*d*^
RER^2^	1.15 [0.10]	1.11 [0.07]	1.15 [0.07]	0.09^*a*^	1.08 [0.07]	1.04 [0.09]	1.05 [0.08]	0.19^*a*^
Maximal exercise intensity (6-20 Borg scale)^2^	17.2 [1.7]	17.4 [1.5]	17.4 [1.8]	0.92^*a*^	17.5 [1.2]	17.2 [1.8]	17.6 [1.1]	0.90^*a*^
HR_*peak*_ (beats/min)^2^	161.7 [13.8]	156.6 [15.9]	159.7 [14.4]	0.66^a^	151.8 [16.4]	154.1 [19.5]	157.3 [14.6]	0.33^*a*^

*^1^results represent number of participants, ^2^results represent mean [±standard deviation]. ^a^Kruskal – Wallis test; ^b^ANOVA; ^c^ln transformed for the analysis, raw data reported; ^d^Pearson Chi-Square; ^e^MoCA was performed at 5 years, ^f^Number of CRF measurements on a treadmill, where a plateau in oxygen uptake (i.e., VO_2__max_) and RER ≥ 1.05 were observed; and those who did not meet the aforementioned criteria (and were therefore referred to as VO_2__peak_); M: men; W: women; P: primary education; S: secondary education; T: tertiary education; Y: yes; N: no; BMI: body mass index; RHR: resting heart rate; DBP: diastolic blood pressure; SBP: systolic blood pressure; HDL: high-density lipoprotein; LDL: low-density lipoprotein; HbA1c: glycated hemoglobin; hsCRP: high sensitivity C-reactive protein; TG: triglycerides; HADS: Hospital Anxiety and Depression Scale; MoCA: Montreal Cognitive Assessment CRF: cardiorespiratory fitness measured as VO_2__peak_; VO_2__peak_: peak oxygen uptake; VO_2__max_: maximal oxygen uptake; RER: maximal respiratory exchange ratio; HR_peak_: peak heart rate.*

**FIGURE 2:**
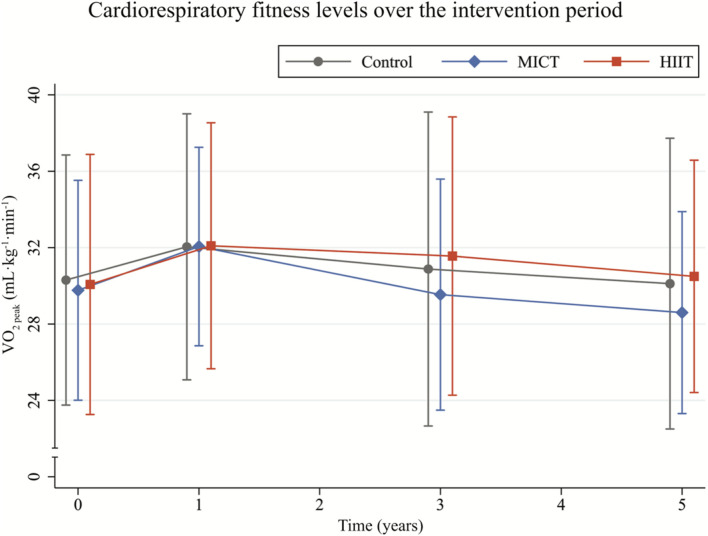
Cardiorespiratory fitness levels, measured as peak oxygen uptake (VO_2__*peak*_), in the Control (gray), high-intensity interval training (HIIT, orange), and moderate-intensity continuous training (MICT, blue) group over time. Whiskers represent one standard deviation below and above the group mean.

During exercise testing, the mean value of RER was 1.09 across the groups and time points while the mean perceived exertion was 17.4 on the Borg scale (Table 2 and Supplementary Table 1). Participants entered the study with a mean VO_2__*peak*_ of 30.11 mL⋅kg^–1^⋅min^–1^, and increased it by about 6.5% in the 1st year of intervention (z = 2.27, *p* = 0.023), followed by a decline to baseline level at the 5-year examination (*z* = −1.91, *p* = 0.056, Figure 2 and Supplementary Table 2). There was no effect of groups on CRF at any time point. Furthermore, the statistical model showed that there was no significant effect of type of measurement (VO_2__*peak*_ versus VO_2__*max*_) on the CRF value (Supplementary Table 2). Additionally, the number of participants who achieved VO_2__*peak*_ versus VO_2__*max*_ was similar across the groups (Table 2 and Supplementary Table 1), which should nullify potential confounding effects of including two types of CRF measurements in the group analysis.

Nevertheless, some VO_2_ measurements did not meet the helping criteria for VO_2__*peak*_ or VO_2__*max*_, and some of the CRF tests were performed on a training bike instead of a treadmill. For a more rigorous assessment of CRF across time and groups and associations to cognition, we formed a supplemental analysis limited to participants who achieved VO_2__*max*_ as defined by a plateau in oxygen uptake on a treadmill and an RER ≥ 1.05. In the participants who achieved VO_2__*max*_, the mean baseline CRF was 30.65 mL⋅kg^–1^⋅min^–1^ and there were no significant differences in CRF between the groups. No effect of time on CRF was present, and the coefficient for the 1-year timepoint was lower in this smaller sample than in the full sample (Supplementary Table 2).

### Adherence to Assigned Exercise and Physical Activity in the High-Intensity Interval Training/Moderate-Intensity Continuous Training/Control Groups

Participants in the HIIT, MICT, and control group adhered well to their prescribed exercise/physical activity program with adherence between 71.4 and 94.6% throughout the intervention (Table 3). There was no significant difference in adherence rate between the groups.

**TABLE 3:** Adherence to the exercise intervention in the control, moderate-intensity continuous training and high-intensity interval training groups.

	Control	MICT	HIIT	*p*-Value
		
	n [%]	n [%]	n [%]	
1 year	38 [90.5%]	16 [76.2%]	23 [74.2%]	0.150
3 years	32 [82.1%]	15 [71.4%]	27 [87.1%]	0.359
5 years	35 [94.6%]	18 [85.7%]	23 [79.3%]	0.173

*MICT: Moderate-intensity continuous training; HIIT: High-intensity interval training; 1 year: 1-year follow-up; 3 years: 3-year follow-up; 5 years: 5-year follow-up; Adherence Control: ≥ 75 min of physical activity per week. Adherence MICT: ≥ 30 min at 11-14 on the Borg scale per week; Adherence HIIT: ≥ 30 min ≥ 15 on the Borg scale per week. Comparison between adherence rates between the three groups at each time point was performed with Pearson Chi-square or Fisher Exact test.*

Average exercise intensity during supervised HIIT and MICT was 88% and 73% of peak heart rate, and the mean rating of perceived exertion was 16.9 and 13.8 on the Borg scale, respectively. As there were no supervised training sessions organized for the control group, this data was only available for the intervention groups.

The average self-reported weekly duration and frequency of exercise sessions, as well as exercise intensity in each group, are described in Table 4 and show that the HIIT group exercised at a higher intensity than the control and MICT groups.

**TABLE 4:** Exercise frequency, duration, and intensity in the control, moderate-intensity continuous training, and high-intensity interval training groups.

	Control	MICT	HIIT	Significant differences
		
	Mean [SD]	Mean [SD]	Mean [SD]	
**Year 1**				
Exercise frequency (sessions per week)	3.0 [1.3]	2.8 [1.3]	3.3 [1.3]	−
Exercise duration (min. per session)	45.7 [14.4]	46.8 [8.2]	47.9 [9.6]	−
Exercise duration per week	140.2 [77.3]	132.3 [75.5]	157.5 [70.9]	−
Exercise intensity (6-20 Borg scale)	13.8 [2.0]	13.6 [0.9]	15.2 [1.5]	C < HIIT***, MICT < HIIT***
**Year 3**				
Exercise frequency (sessions per week)	3.0 [1.7]	2.9 [1.2]	3.4 [1.4]	−
Exercise duration (min. per session)	46.1 [14.0]	49.0 [10.0]	47.4 [12.0]	−
Exercise duration per week	146.9 [86.7]	147.8 [53.8]	157.7 [72.4]	−
Exercise intensity (6-20 Borg scale)	13.2 [2.6]	13.4 [0.9]	15.5 [1.3]	C < HIIT***, MICT < HIIT***
**Year 5**				
Exercise frequency (sessions per week)	3.3 [1.5]	2.8 [1.3]	3.2 [1.4]	−
Exercise duration (min. per session)	48.6 [14.2]	50.1 [10.0]	44.4 [13.1]	−
Exercise duration per week	170.6 [93.2]	141.1 [75.3]	138.5 [75.9]	−
Exercise intensity (6-20 Borg scale)	13.4 [1.7]	12.5 [2.1]	15.0 [1.4]	C < HIIT***, MICT < HIIT***

MICT: Moderate Intensity Continuous Training; HIIT: High Intensity Interval Training. ***p ≤ 0.001.

Table 5 shows the self-reported frequencies of performing different types of activities in each group throughout the intervention. HIIT participants reported that they more often, compared to the control group: cycle at 1 year (*z* = 1.90, *p* = 0.029), use fitness centers at 5 years (*z* = 1.68, *p* = 0.047), and swim at 3 years (z = 2.71, *p* = 0.003). Compared to the MICT group, HIIT participants reported cycling more often at 1 year (*z* = 2.42, *p* = 0.008), swimming at 3 years (*z* = 3.55, *p* < 0.001), and using fitness centers at 5 years (*z* = 2.83, *p* = 0.002). No differences between the control group and the MICT group were observed here.

**TABLE 5:** Exercise types and weekly frequency in the control, moderate-intensity continuous training, and high-intensity interval training groups throughout the intervention.

	Control	MICT	HIIT	Significant differences
	Mean [SD]	Mean [SD]	Mean [SD]	
**Year 1**				
Walking^1^	2.34 [1.20]	2.47 [0.95]	2.47 [0.95]	−
Cycling	0.75 [0.93]	1.04 [2.18]	1.74 [2.09]	MICT < HIIT**, Control < HIIT*
Swimming	0.27 [0.49]	0.21 [0.30]	0.51 [0.76]	−
Skiing (in winter)	0.72 [1.1]	0.71 [1.00]	0.73 [0.92]	−
Fitness center	0.99 [1.2]	0.96 [1.17]	1.47 [1.35]	−
Organized sports	0.15 [0.39]	0.27 [0.49]	0.32 [0.59]	−
Other activities	0.22 [0.64]	0.19 [0.38]	0.53 [0.82]	−
**Year 3**				
Walking^1^	2.26 [1.26]	1.97 [1.36]	2.68 [1.88]	−
Cycling	0.77 [1.15]	1.01 [2.02]	1.49 [1.90]	−
Swimming	0.28 [0.60]	0.09 [0.12]	0.52 [0.65]	Control < HIIT**, MICT < HIIT***
Skiing (in winter)	0.68 [1.10]	0.87 [1.70]	0.72 [0.95]	−
Fitness center	0.87 [1.13]	0.63 [0.83]	1.30 [1.17]	−
Organized sports	0.30 [0.76]	0.27 [0.39]	0.56 [0.98]	−
Other activities	0.50 [0.68]	0.56 [0.65]	0.49 [0.62]	−
**Year 5**				
Walking^1^	2.20 [1.32]	1.81 [1.00]	2.26 [1.63]	−
Cycling	0.74 [1.34]	0.39 [0.78]	1.60 [2.16]	−
Swimming	0.31 [0.89]	0.08 [0.12]	0.43 [0.70]	−
Skiing (in winter)	0.55 [0.89]	0.21 [0.33]	0.49 [0.80]	−
Fitness center	0.86 [1.28]	0.32 [0.64]	1.19 [1.13]	Control < HIIT*, MICT < HIIT**
Organized sports	0.35 [1.06]	0.42 [0.80]	0.51 [0.83]	−
Other activities	0.40 [0.51]	0.61 [0.62]	0.44 [0.40]	−

*p < 0.050, **p ≤ 0.010, ***p ≤ 0.001. MICT: Moderate Intensity Continuous Training; HIIT: High-Intensity Interval Training.

*^1^Walking as a means of transport, recreational walking, and hiking in nature.*

*Values represent the self-reported weekly frequency of listed activities.*

### Performance on the Cognitive Tests in the Control, High-Intensity Interval Training, and Moderate-Intensity Continuous Training Groups

#### Main Models

**FIGURE 3:**
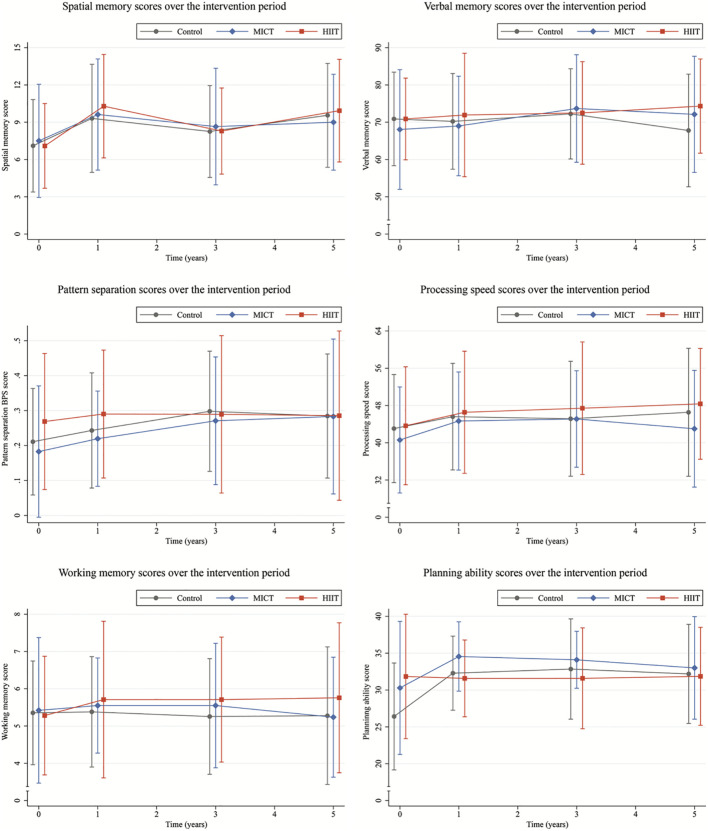
Performance of the Control (gray), moderate-intensity continuous training (MICT, blue), and high-intensity interval training (HIIT, orange) groups on the cognitive tests over time. Whiskers represent one standard deviation below and above the group mean.

Figure 3 shows the performance raw scores on all cognitive tests over time for each group. Regardless of group assignment, participants experienced no significant decline over the 5 years on the cognitive measures. Spatial memory, pattern separation, and planning ability improved at one or more time points during the intervention.

Model 1 (Table 6) showed no significant group^∗^time interaction, meaning that there were no effects of HIIT or MICT intervention on any cognitive test at any time point, besides a negative effect of HIIT on planning ability at 3 years (*z* = −2.07, *p* = 0.039). Still, a higher CRF during the intervention was associated with higher processing speed, independent of the group (*z* = 2.68, *p* = 0.007). In Model 2 (Table 7), which included the analysis of group^∗^time interaction in the context of change in CRF on cognition, no association between HIIT or MICT intervention on any cognitive test was present at any time point. Across all groups, an increase in CRF during the intervention was associated with better working memory (*z* = 2.11, *p* = 0.035). Model 3 (Table 8) showed that higher baseline CRF predicted better cognitive function at later time points for verbal memory (after 3 years: *t* = 2.07, *p* = 0.042), pattern separation (after 1 year: *t* = 2.00, *p* = 0.049; and 5 years: *t* = 2.09, *p* = 0.040), and processing speed (after 1 year: *t* = 3.01, *p* = 0.003; 3 years: *t* = 2.93, *p* = 0.004; and 5 years: *t* = 2.49, *p* = 0.015).

**TABLE 6:** Results of the linear mixed model (Model 1) assessing group *time interaction, cardiorespiratory fitness (CRF), and time effects on cognitive test performance in the high intensity interval training group and moderate intensity continuous training group compared to the control group.

	Spatial memory	Verbal memory	Pattern separation	Processing speed	Working memory	Planning ability
	
Predictors	Coef. [CI]	Coef. [CI]	Coef. [CI]	Coef. [CI]	Coef. [CI]	Coef. [CI]
CRF	0.00 [−0.02,0.02]	0.01 [−0.01,0.03]	0.01 [−0.01,0.03]	0.02** [0.01,0.04]	0.02 [−0.00,0.04]	0.01 [−0.01,0.03]
1 year	0.57***# [0.28,0.85]	−0.12 [−0.37,0.13]	0.14 [−0.14,0.43]	0.19 [−0.02,0.41]	0.05 [−0.19,0.30]	0.57**# [0.22,0.91]
3 years	0.22 [−0.09,0.52]	0.09 [−0.17,0.36]	0.52***# [0.21,0.83]	0.19 [−0.05,0.42]	−0.00 [−0.27,0.26]	0.81***# [0.44,1.18]
5 years	0.52**# [0.20,0.83]	−0.13 [−0.41,0.15]	0.35* [0.03,0.66]	0.20 [−0.05,0.44]	−0.09 [−0.36,0.18]	0.72***# [0.32,1.12]
MICT*1 year	−0.08 [−0.54,0.38]	0.04 [−0.38,0.45]	−0.17 [−0.63,0.30]	−0.05 [−0.41,0.32]	−0.06 [−0.48,0.36]	0.15 [−0.34,0.65]
MICT*3 years	0.01 [−0.49,0.51]	0.27 [−0.17,0.72]	−0.45 [−0.96,0.06]	0.08 [−0.32,0.48]	0.15 [−0.30,0.60]	−0.10 [−0.64,0.45]
MICT*5 years	−0.20 [−0.69,0.30]	0.15 [−0.30,0.61]	−0.09 [−0.59,0.41]	−0.12 [−0.51,0.28]	−0.01 [−0.46,0.43]	−0.19 [−0.74,0.35]
HIIT*1 year	0.09 [−0.31,0.50]	0.13 [−0.24,0.50]	0.06 [−0.36,0.47]	−0.11 [−0.43,0.21]	0.07 [−0.29,0.43]	−0.27 [−0.70,0.16]
HIIT *3 years	0.03 [−0.39,0.46]	−0.05 [−0.44,0.33]	−0.35 [−0.78,0.09]	0.01 [−0.33,0.35]	0.21 [−0.17,0.59]	−0.49* [−0.96,−0.03]
HIIT*5 years	0.27 [−0.18,0.72]	0.24 [−0.17,0.65]	−0.00 [−0.46,0.46]	0.05 [−0.31,0.41]	0.30 [−0.10,0.70]	−0.35 [−0.86,0.16]
N	340	313	338	338	336	293

*p < 0.050, **p ≤ 0.010, ***p ≤ 0.001; #: effect still significant after Holm-Bonferroni correction. Coef.: coefficients; CI: confidence intervals; CRF: cardiorespiratory fitness measured as VO_2__peak_; MICT: Moderate Intensity Continuous Training; HIIT: High Intensity Interval Training; 1 year: 1-year follow-up; 3 years: 3-year follow-up; 5 years: 5-year follow-up; N: number of observations. Besides the variables shown in the table, the model controlled for age at inclusion, sex, and education.

**TABLE 7:** Results of the linear mixed model (Model 2) assessing group*time interaction, change in cardiorespiratory fitness, and time effects on cognitive test performance during the intervention in the high intensity interval training group and moderate intensity continuous training group compared to the control group.

	Spatial memory	Verbal memory	Pattern separation	Processing speed	Working memory	Planning ability
	
Predictors	Coef. [CI]	Coef. [CI]	Coef. [CI]	Coef. [CI]	Coef. [CI]	Coef. [CI]
CRF change	−0.01 [−0.04,0.02]	0.01 [−0.01,0.04]	−0.02 [−0.05,0.01]	0.01 [−0.02,0.03]	0.03* [0.00,0.05]	−0.01 [−0.04,0.02]
1 year	0.58*** [0.30,0.86]	−0.13 [−0.38,0.12]	0.19 [−0.08,0.47]	0.21 [−0.01,0.43]	0.05 [−0.20,0.29]	0.58**# [0.23,0.93]
3 years	0.21 [−0.09,0.52]	0.09 [−0.17,0.35]	0.53***# [0.23,0.83]	0.18 [−0.05,0.42]	−0.00 [−0.27,0.27]	0.81***# [0.44,1.18]
5 years	0.49** [0.18,0.81]	−0.14 [−0.41,0.14]	0.32* [0.01,0.63]	0.17 [−0.07,0.42]	−0.07 [−0.35,0.20]	0.69***# [0.29,1.10]
MICT*1 year	−0.03 [−0.50,0.44]	0.20 [−0.22,0.61]	−0.22 [−0.69,0.25]	0.01 [−0.37,0.39]	−0.02 [−0.45,0.41]	0.24 [−0.29,0.76]
MICT*3 years	0.04 [−0.46,0.54]	0.32 [−0.12,0.76]	−0.47 [−0.97,0.02]	0.11 [−0.29,0.51]	0.16 [−0.29,0.61]	−0.09 [−0.63,0.46]
MICT*5 years	−0.07 [−0.58,0.43]	0.20 [−0.25,0.65]	−0.04 [−0.54,0.46]	−0.01 [−0.42,0.40]	−0.01 [−0.47,0.45]	−0.05 [−0.62,0.52]
HIIT*1 year	0.07 [−0.34,0.47]	0.11 [−0.25,0.47]	0.06 [−0.34,0.47]	−0.10 [−0.43,0.22]	0.06 [−0.30,0.43]	−0.27 [−0.70,0.17]
HIIT *3 years	0.04 [−0.39,0.46]	−0.04 [−0.42,0.34]	−0.37 [−0.80,0.05]	0.03 [−0.32,0.37]	0.17 [−0.21,0.56]	−0.47 [−0.94,0.01]
HIIT*5 years	0.26 [−0.19,0.72]	0.24 [−0.16,0.64]	−0.08 [−0.53,0.37]	0.08 [−0.29,0.44]	0.32 [−0.08,0.73]	−0.34 [−0.86,0.18]
N	333	309	331	331	329	286

*p < 0.050, **p ≤ 0.010, ***p ≤ 0.001; #: effect still significant after Holm-Bonferroni correction. Coef.: coefficients; CI: confidence intervals; CRF, cardiorespiratory fitness, change: change in VO_2peak_ between each time point; MICT: Moderate Intensity Continuous Training; HIIT: High Intensity Interval Training; 1 year: 1-year follow-up; 3 years: 3-year follow-up; 5 years: 5-year follow-up; N: number of observations. Besides the variables shown in the table, the model controlled for age at inclusion, sex, and education.

**TABLE 8:** Results of the linear regression (Model 3) assessing if CRF at baseline could predict cognitive performance after 1-, 3-, and 5 years of intervention.

	Spatial memory	Verbal memory	Pattern separation	Processing speed	Working memory	Planning ability
	
Time point	Coef. [CI]	Coef. [CI]	Coef. [CI]	Coef. [CI]	Coef. [CI]	Coef. [CI]
1 year	0.03 [−0.01, 0.06]	0.02 [−0.02, 0.05]	0.04* [0.00, 0.07]	0.05** [0.02, 0.08]	0.02 [−0.02, 0.05]	0.03 [−0.01, 0.07]
3 years	0.02 [−0.02,0.06]	0.03* [0.00,0.07]	0.03 [−0.01,0.07]	0.05** [0.02,0.09]	0.03 [−0.01,0.06]	0.03 [−0.01,0.07]
5 years	0.03 [−0.00, 0.07]	0.03 [−0.01, 0.07]	0.04* [0.00, 0.07]	0.04* [0.01, 0.08]	0.03 [−0.01, 0.06]	0.02 [−0.02, 0.06]

A separate regression was run for each time point. *p < 0.050, **p ≤ 0.010, ***p ≤ 0.001. Coef.: coefficients; 95% CI: confidence intervals; CRF: cardiorespiratory fitness; 1 year: 1-year follow-up; 3 years: 3-year follow-up; 5 years: 5-year follow-up. Besides the variables shown in the table, the model controlled for age at the time of testing, sex, and education. No tests were significant after Holm-Bonferroni correction.

#### Supplementary Analysis

When only participants who achieved VO_2__*max*_ were included in Model 1 (Supplementary Table 3), a negative interaction between the MICT group at 3 years and pattern separation was observed (*t* = −2.21, *p* = 0.027). As in the main model, higher CRF during the intervention was associated with higher processing speed (*t* = 2.70, *p* = 0.007).

In Supplementary Model 2 (Supplementary Table 4), a negative interaction between the MICT group at 3 years and pattern separation was also observed (*t* = −2.13, *p* = 0.033). An increase in CRF was associated with worse pattern separation (*z* = −2.56, *p* = 0.010).

In Supplementary Model 3 (Supplementary Table 5), higher baseline CRF predicted better verbal memory (after 3 years: *t* = 2.58, *p* = 0.014; and 5 years: *t* = 2.07, *p* = 0.048) and processing speed (after 1 year: *t* = 2.56, *p* = 0.014; and 3 years: *t* = 3.31, *p* = 0.002).

## Discussion

The 5-year RCT with older adults randomized into HIIT, MICT, or a control group following national guidelines for physical activity, did not find that HIIT at any time point during the 5-year intervention improved performance on the cognitive tests relative to the control group. Contrary to our hypothesis, HIIT was associated with a lower planning ability score at 3 years (Model 1). Nevertheless, in line with our prediction, we demonstrated several positive relationships between CRF and cognition which were consistent across the different statistical models. Higher CRF during the intervention was associated with faster processing speed. A positive change in CRF during the intervention was associated with an improvement in working memory, and a higher baseline CRF predicted better processing speed, pattern separation, and verbal memory at later time points. When investigating only the participants who achieved VO_2__*max*_, the results were quite similar, but some notable differences were uncovered including a negative effect of increasing CRF on pattern separation (Supplementary Model 2). Additionally, a negative interaction between MICT and the 3-year time point on pattern separation was observed (Supplementary Models 1, 2). Overall, CRF rather than the prescribed exercise regime was shown to enhance cognitive aging thus supporting the cardiovascular fitness hypothesis, but not for all cognitive abilities.

### Intervention Effects on Cognition in High-Intensity Interval Training, Moderate-Intensity Continuous Training, and Control Groups

Contrary to our hypothesis, we did not uncover a positive effect of being in the HIIT group on cognition. Rather we uncovered a negative effect of the HIIT intervention, relative to the control group, on planning ability at the 3-year follow-up. When including only VO_2__*max*_ measurements in the model, MICT was negatively associated with pattern separation at 3 years. The control group thus emerged as having a slightly better cognitive outcome during the intervention. The better performance in the control group relative to HIIT in the main model could be a spurious finding stemming from regression toward the mean as the HIIT group entered the study with (statistically insignificant) higher scores in planning ability and then scored more similarly to the control group during the intervention. In turn, the weaker pattern separation performance of the MICT group at 3 years could be incidental and due to the low number of participants in the MICT group (*n* = 8) in Supplementary Models. This is supported by the fact the effect is not present at the other time points.

Participants in the three groups exercised or followed the national physical activity guidelines in line with the specification of their allocated group. Both the HIIT and MICT groups exercised at the prescribed heart rates and ratings of perceived exhaustion throughout the intervention. The groups did not differ when it comes to the frequency or the duration of exercise sessions (Table 4), and both the supervised training groups and the control group adhered well to their described level of exercise or the physical activity guideline as defined in the RCT (cf. Table 3). The HIIT group was further shown to be cycling, swimming, and exercising in fitness centers more compared to both the control and MICT groups (Table 5). Since we did not uncover any notable differences related to the intervention group on cognition over time, and the participants in all groups remained cognitively stable throughout the 5 years, our results support that any physical activity and/or exercise at HIIT and/or MICT levels is beneficial for maintaining cognitive abilities in older adults.

### Cardiorespiratory Fitness Across the Intervention Groups

Despite differences in training intensity and good adherence throughout the intervention period to prescribed activities, all groups had similar mean CRF levels during the 5 years and a significant, but modest increase in CRF of 6.5% (or 2 mL⋅kg^–1^⋅min^–1^) during the 1st year in this study, followed by a decrease to baseline levels at 5 years. When assessing only VO_2__*max*_ observations, no significant increase in CRF was observed with time nor any group-by-time interactions. The lack of a CRF increase in the VO_2__*max*_ sample shows that it is difficult to increase CRF in already physically active older adults, even when participants adhere well to their prescribed activities.

If cognitive health is linked to CRF, as suggested by the cardiovascular fitness hypothesis (Voss, 2016), the lack of group differences in CRF over time could explain the lack of positive group-by-time interactions in our study.

Previous studies reporting positive effects of specific exercise interventions on cognitive abilities have usually included participants with peak/max VO_2_ around 20 mL⋅kg^–1^⋅min^–1^ at inclusion and compared one sedentary control group with one group performing aerobic exercise for 6 weeks to 24 months (Madden et al., 1989; Panton et al., 1990; Moul et al., 1995; Kramer et al., 2002; Ellis et al., 2009; Ngandu et al., 2015; Jonasson et al., 2017; Kovacevic et al., 2020). The participants’ age varied from 60 to over 80 years, and a clinical diagnosis (e.g., mild cognitive impairment, depression) was frequent (Etnier et al., 1997; Colcombe and Kramer, 2003; Smith et al., 2010). The inclusion of only cognitively healthy participants could potentially mask beneficial intervention effects in a specific group akin to those uncovered in other studies (Young et al., 2015). Overall, our participants had higher CRF at inclusion (30.11 mL⋅kg^–1^⋅min^–1^) than in earlier interventional studies, but comparable to values found in the same age group in another Norwegian general population (Loe et al., 2013). Our sample should therefore be representative of older Norwegians. The increase in CRF in our study was only observed during the 1st year and was within the lower range of what has been previously reported, e.g., 5.1 and 20.4% (Blumenthal et al., 1989; Panton et al., 1990; Moul et al., 1995; Kramer et al., 2002; Jonasson et al., 2017; Kovacevic et al., 2020). Since even a small increase in CRF is beneficial for somatic health, including increases in life expectancy, this increase is not negligible (Stensvold et al., 2020). Furthermore, there was no significant decline in CRF across the 5 years. Previous studies suggest a decline of approximately 3 mL⋅kg^–1^⋅min^–1^ in this age range in Norwegian general population samples (Aspenes et al., 2011; Loe et al., 2013).

Taken together, our results indicate that it is more difficult to achieve a large increase in CRF in healthy older adults with a high baseline CRF, also in a HIIT group, but it was possible to stave of CRF decline which may be as important in older age as improving it at the higher end of the CRF range.

### Cardiorespiratory Fitness and Cognition in Older Adults

Although we did not find a positive effect of HIIT or MICT compared to following the national physical activity guidelines on cognition during the intervention, we did demonstrate some of the predicted beneficial effects of CRF level during the intervention and change in CRF on cognition in older adults, as well as the importance of baseline CRF on future cognition. These findings are in line with previous studies on the relationship between CRF and cognition although it should be noted that the literature is inconsistent (Angevaren et al., 2008; Smith et al., 2010; Young et al., 2015; Gomes-Osman et al., 2018). Our main and supplemental analyses revealed a positive relationship between CRF and processing speed throughout the intervention. We also uncovered the beneficial effect of increasing CRF on working memory. Furthermore, higher CRF at baseline predicted better verbal memory, pattern separation, and processing speed at later time points. None of our analyses showed the expected positive relationship between CRF and planning ability, or any relationships between CRF and spatial memory.

The beneficial effect of aerobic exercise on processing speed is among the most consistently reported in interventional studies (Angevaren et al., 2008; Smith et al., 2010; Gomes-Osman et al., 2018). Still, we did not find an effect of HIIT or MICT on processing speed. Processing speed is an ability sensitive to environmental influences even in old age (Rebok et al., 2014; Reynolds and Finkel, 2015), but based on our results the type of aerobic training or physical activity appears to be less important than entering into old age with a high CRF and maintaining it. This is based on the lack of a positive effect of increasing CRF (Model 2) on processing speed, but a positive association of CRF on processing speed throughout the intervention (Model 1) and baseline CRF⋅s positive association with future processing speeds (Model 3). Indeed, depending on the model, the fittest older adults of our cohort are (or will be) 22 to 57% faster than the least fit. This relationship was even stronger when only looking at participants who reached VO_2__*max*_.

The relationship between pattern separation and CRF has been studied in aerobic exercise intervention studies in humans but even more often in rodents and usually report improved pattern separation ability (Creer et al., 2010; Bolz et al., 2015; Vivar et al., 2016; Suwabe et al., 2018; Bernstein and McNally, 2019). The positive effect is believed to be mediated by CRF-induced increases in neurogenesis (Sahay et al., 2011; Vivar et al., 2016). The results of our study are not in line with this work as we found no improvement in pattern separation in response to increasing CRF or belonging to the HIIT or MICT group. On the contrary, our supplementary analyses demonstrated negative relationships between pattern separation and MICT group at 3 years, as well as between pattern separation and increasing CRF. While the group effect is likely to be a spurious finding, the negative relationship between pattern separation and increased CRF is unexpected. This negative finding could be due to assessment of long-term effects in this study, in contrast to short-term or acute effects examined in previous rodent work. Since the existence of neurogenesis in adult human hippocampus is controversial (Sorrells et al., 2021), it is also possible that humans and rodents experience species specific responses to aerobic exercise. It should also be noted that the mechanisms by which the CRF-induced increases in neurogenesis mediate improvements in pattern separation in rodents remain unknown (Johnston et al., 2016). Thus, it may be that the relationship does not persist in the long-term, if at all (Becker, 2017; Sorrells et al., 2021). Nevertheless, we found baseline CRF to predict better pattern separation, which, to our knowledge, has not been shown before. According to Model 3, the fittest older adults at baseline were about twice as effective as the least fit participants at distinguishing between similar, new, and old items after 3 and 5 years.

The relationship between verbal memory and CRF is debated, with some studies finding a positive relationship (Barnes et al., 2003; Etnier et al., 2007; Smith et al., 2010), while others do not (Stroth et al., 2008; Langlois et al., 2013). According to estimates in our Model 3, the least fit persons at baseline could memorize 11.5 items from a list of 16 words at 3 years, and the fittest could remember about 13.5 items. Surprisingly, the effect persisted and was even more pronounced when only examining participants who achieved VO_2__*max*_, suggesting that VO_2__*max*_ is a better predictor of verbal abilities than VO_2__*peak*_.

When investigating working memory performance, we observed that it increased with an increase in CRF during the intervention, even if only significant in the main analysis (Model 2). This was in line with our prediction providing direct support to the cardiovascular fitness hypothesis albeit consistent for only one cognitive ability. Although significant, the effect on working memory was unfortunately relatively small. Based on our results, a change in VO_2__*peak*_ of 12 mL⋅kg^–1^⋅min^–1^ (i.e., the difference between a person from the 5th percentile and a person from the 95th percentile in our study) accounted for an ability to remember approximately half a digit more. However, in cohorts of individuals that are less fit than in our study, it may be possible to gain a meaningful increase of working memory for everyday life by improving CRF. In general, the effect of change in CRF on working memory as well as the association between CRF and working memory are inconsistent in the literature. Some studies using digit span tests similar to ours report a positive effect (Kramer et al., 2002; Voss et al., 2013), but no effect for spatial working memory (Erickson et al., 2011). Earlier reviews reported no association between exercise interventions and working memory, even in interventions increasing CRF (Smith et al., 2010; Young et al., 2015), but a recent systematic review found a significant positive effect on working memory (Xiong et al., 2020). It should be noted that we did not find a significant association between CRF and working memory in the Supplementary Models or the linear regression analyses and only a trend in Model 1. Thus, our results support that increase in CRF is beneficial for working memory, but only to a minor extent.

The performance on all cognitive tests was stable throughout the 5-year period. This is a somewhat surprising finding in the context of a recent cognitive trajectories study that suggested cognitive aging trends of linear or accelerating decline from early adulthood (Salthouse, 2019). Still, other studies have shown a considerable heterogeneity of cognitive trajectories, including no decline, dependent on several factors (Mungas et al., 2010; Yu et al., 2020). The relative stability in cognitive abilities as measured long-term in older adults has been shown previously (Lundervold et al., 2014; Mistridis et al., 2015; Idland et al., 2020) and is especially pronounced in cohorts of highly educated, active, healthy individuals with a high socioeconomic status, exactly the kind of persons who opt-in and remain in intervention studies like ours (Tranel et al., 1997; Compton et al., 2000; Hickman et al., 2000; Lezak et al., 2004; Nyberg and Pudas, 2019). The results in this study are thus from cognitively well-functioning older adults, with a low risk of developing cognitive impairments. Still, we were able to uncover significant positive associations between CRF and multiple cognitive abilities, demonstrating that CRF plays a part in cognitive aging, even in healthy older adults who can be described as aging successfully.

Taken together our results show a connection between CRF and certain cognitive abilities. Processing speed has the strongest association with the CRF at the time of testing and baseline CRF predicted better future performance. Working memory, on the other hand, benefited from an increase in CRF. Baseline CRF predicted better pattern separation at later time points but increasing CRF during the intervention was related to poorer pattern separation, which became significant when investigating the participants who achieved VO_2__*max*_. Some differences were found between the models that included all the CRF measurements versus those that only included VO_2__*max*_, but the results were generally consistent across the different statistical models. This supports their relevance, bearing in mind that we evaluated the results not corrected for multiple comparisons. Our results suggest that cognitive abilities are differentially modifiable by different aspects of CRF. In practical terms, this means that entering old age with a high CRF and maintaining or increasing it throughout old age provides the overall best effect across cognitive abilities.

## Strengths and Limitations

This study offers a fuller picture of the long-term effects of an exercise intervention on cognitive abilities than previous, shorter RCTs. The main strengths of this study were: a general population sample, the implementation of three groups, including an active control group and two supervised exercise groups training at different intensities, information on adherence to the prescribed group⋅s exercise regime or national physical activity guidelines, several follow-up time points with standardized clinical and cognitive assessments, robust statistical models, and the 5-year duration.

Most previous RCTs lasted from 6 weeks (Scherder et al., 2005) to 2 years (Ngandu et al., 2015). A 5-year intervention has so far not been performed at any age and allowed us to investigate long-term instead of short-term effects on cognition, which are often difficult to interpret due to practice effects on cognitive tests. The sample size was calculated based on a lower incidence of decline in performance on cognitive tests across the 5 years in the supervised training groups compared to the control group in which performance was expected to decline between 33 and 54% (Dodge et al., 2006; Howieson et al., 2008; Terrera et al., 2010; Yaffe et al., 2010) leading to group sizes between 9 and 19 participants required to find differences with an alpha of 0.05 and power of 80%. Unexpectedly, all groups included in our study appeared to be very cognitively stable. This could be a selection bias (see above) or related to the fact that all groups were very physically active although at different intensities and activities. In fact, despite the lack of supervised training, the control group appeared to train with a very similar frequency and intensity to the MICT group, while also equally often engaging in various types of physical activities, which could have masked potential group effects. Sample sizes between 10 and 100 participants are needed to detect cognitive benefits that can be derived from early exercise intervention studies (Williams and Lord, 1997; Marmeleira et al., 2009; Klusmann et al., 2010; Kim et al., 2011). Since this study was planned, many new studies have been performed and the effect sizes uncovered in meta-analyses of exercise intervention were smaller than the earlier studies suggested (Smith et al., 2010). Nevertheless, we were able to uncover effects of CRF and changes in CRF on cognition in our sample.

The use of VO_2__*peak*_ as a CRF measurement in the study could be a weakness as there is contention surrounding its validity (Poole and Jones, 2017). Yet, other studies show that there is not much of a difference using VO_2__*max*_ or VO_2__*peak*_ (Whipp, 2010). To address the issue, we provided supplementary analyzes that included only VO_2__*max*_ measurements. The statistical results obtained with VO_2__*max*_ suggested that VO_2__*max*_ could be a better predictor of cognitive abilities, demonstrated by uncovering a new effect that was not significant in the main analysis, more prominent coefficients and smaller CIs for CRG and changes in CRF, and baseline CRF predictions of future cognitive functions despite considerably lower sample size. Still, limiting the analysis to VO_2__*max*_ is not without its drawbacks. Namely, the lower sample size significantly reduced the statistical power of the models, especially those including comparisons between groups. It is also possible that excluding VO_2__*peak*_ measurements and measurements taken on a bike introduced additional bias, as factors preventing participants from reaching VO_2__*max*_ or testing on a treadmill could have also been related to their cognitive functioning. More studies are needed to verify this as there are not many studies that use VO_2__*max*_ in older adults currently.

There is a bias in who is interested in participating in scientific studies and remain in them for the entire duration of the intervention. The participants in this study were healthy (see Table 1) and remained so throughout the study. The two participants who died did so due to cancer. The participants who remained in the study for 5 years had a higher processing speed than those dropping out. The participants maintained their cognitive abilities throughout the intervention, and none became demented as reflected by the MoCA scores. As such our results do not follow the pattern of progressive decline in many cognitive domains shown in norms and cross-sectional studies of cognitive aging (Bopp and Verhaeghen, 2005; Rönnlund et al., 2005; Singh-Manoux et al., 2012).

It should be noted that we based our interpretation of the results on the *p*-Values not corrected for multiple comparisons, as per protocol. Correcting for multiple comparisons is highly debated among statisticians (Rothman, 1990, 2014). Readers of this article in favor of mathematical corrections should consult the tables to check which results remain significant after the correction as neither CRF nor training group^∗^time interaction associations survived correction for multiple comparisons. They should also consider the confidence intervals and coefficients provided for the models when evaluating the relative impact of the intervention group, CRF, and change in CRF.

## Conclusion

Our results support the cardiovascular fitness hypothesis as we demonstrated that higher CRF and increasing CRF benefited multiple, but not all, cognitive abilities in older adults. The HIIT or MICT intervention did not appear to enhance performance on cognitive abilities above the control condition which was 30 min of moderate-intensity physical activity almost every day. In conclusion, going into retirement with good CRF and maintaining it after the age of 70 preserved cognition, but the mode of exercise appeared less important. Increasing CRF in old age can be good for some abilities but potentially bad for others.

## Data Availability Statement

Because of privacy concerns and state regulations, the ethical and governance approvals for this study do not allow the data to be made available in a public repository. Data in this manuscript can be accessed by qualified investigators after ethical and scientific review (to ensure the data are being requested for valid scientific research) and must comply with the European Union General Data Protection Regulations (GDPR), Norwegian laws and regulations, and NTNU regulations. The completion of a material transfer agreement (MTA) signed by an institutional official will be required. Requests to access the datasets should be directed to AH (asta.haberg@ntnu.no).

## Ethics Statement

The studies involving human participants were reviewed and approved by Regional Committee for Medical Research Ethics, Central Norway (2012/849). The patients/participants provided their written informed consent to participate in this study.

## Author Contributions

DRS contributed to the data quality control and organization, performed the statistical analysis, drafting, and revision of the manuscript. TH performed the cognitive data collection, oversaw cognitive data QC and organization, statistical analyses, and revision of the manuscript. HR contributed to the statistical analysis, figures, and revised the manuscript. LR performed the clinical data collection, QCed clinical and CRF data used in the manuscript, and revised the manuscript. UW did the co-PI of the RCT generation 100 study, responsible for exercise, health, clinical data collection, and revised the manuscript. DS contributed to the PI of the RCT generation 100 study, responsible for exercise, health, and clinical data collection, and revised the manuscript. AH contributed to the PI of cognitive substudy in generation 100, organized the data collection, collected the data, carried out the statistical analysis, drafted the manuscript, and revised the manuscript. All authors contributed to the article and approved the submitted version.

## Conflict of Interest

The authors declare that the research was conducted in the absence of any commercial or financial relationships that could be construed as a potential conflict of interest.

## Publisher’s Note

All claims expressed in this article are solely those of the authors and do not necessarily represent those of their affiliated organizations, or those of the publisher, the editors and the reviewers. Any product that may be evaluated in this article, or claim that may be made by its manufacturer, is not guaranteed or endorsed by the publisher.
