# Supplementary material for: 5 Years of Exercise Intervention Did Not Benefit Cognition Compared to the Physical Activity Guidelines in Older Adults, but Higher Cardiorespiratory Fitness Did. A Generation 100 Substudy
Source: Front Aging Neurosci. 2021 Nov 16;13:742587. doi: 10.3389/fnagi.2021.742587 (PMC8637860; doi:10.3389/fnagi.2021.742587)
Supplement: Supplementary file 1 [file Table_1.docx]

*Supplementary Table 1.* *Demographics, physical measures, and clinical variables for the control group, the Moderate Intensity Continuous training group (MICT), and the High Intensity Interval Training group (HIIT) at the 1- and 3-year follow-ups.*

|  | **One-year follow-up** | | | |  | **Three-year follow-up** | | | |
| --- | --- | --- | --- | --- | --- | --- | --- | --- | --- |
|  | Control | MICT | HIIT | p-value |  | Control | MICT | HIIT | *p*-value |
| **Sex (M/W)^1^** | 20/22 | 10/11 | 17/14 | .80^d^ |  | 19/20 | 10/11 | 17/14 | .84^d^ |
| **Age (years)^2^** | 73.6 [1.8] | 73.8 [1.8] | 73.9 [2.1] | .89^a^ |  | 75.4 [1.8] | 75.5 [1.8] | 75.6 [2.1] | .88^a^ |
| **Education (P/S/T)^1^** | 2/13/27 | 3/3/15 | 2/6/22 | .42^d^ |  | 2/12/25 | 3/3/15 | 2/6/22 | .47^d^ |
| **Living alone (Y/N)^1^** | 14/26 | 6/15 | 8/23 | .69^d^ |  | 14/23 | 4/14 | 8/23 | .40^d^ |
| **Current smoker (Y/N)^1^** | 4/37 | 1/20 | 3/28 | .78^d^ |  | 4/33 | 1/17 | 2/29 | .73^d^ |
| **Height (cm)^2^** | 168.2 [9.2] | 170.8 [8.0] | 169.9 [8.7] | .26^b^ |  | 167.6 [9.4] | 171.0 [8.1] | 170.1 [8.7] | .22^b^ |
| **Weight (kg)^2^** | 71.9 [13.9] | 75.2 [10.2] | 74.4 [12.9] | .58^b^ |  | 72.2 [12.6] | 76.0 [11.4] | 75.7 [13.0] | .48^b^ |
| **Waist circumference (cm)^2^** | 91.0 [12.8] | 92.4 [9.5] | 90.9 [10.4] | .76^b^ |  | 93.7 [11.6] | 94.3 [10.2] | 93.1 [10.0] | .87^b^ |
| **Muscle mass (%)^2^** | 27.6 [5.7] | 29.0 [4.8] | 29.1 [5.4] | .23^b^ |  | 27.1 [5.8] | 29.1 [4.8] | 29.1 [5.3] | .09^b^ |
| **Fat (%)^2^** | 29.4 [7.7] | 29.4 [7.9] | 27.3 [7.6] | .57^b^ |  | 30.6 [7.9] | 29.7 [8.0] | 29.5 [6.6] | .92^b^ |
| **BMI (kg/m^2^)^2^** | 25.3 [3.8] | 25.8 [3.7] | 25.5 [3.1] | .88^b^ |  | 25.6 [3.3] | 26.0 [3.9] | 26.1 [3.0] | .87^b^ |
| **RHR (beats/min)^2^** | 64.3 [10.0] | 65.3 [8.0] | 60.8 [7.1] | .16^b^ |  | 61.2 [8.9] | 63.6 [10.5] | 59.9 [6.8] | .32^b^ |
| **DBP right (mmHg)^2^** | 73.3 [7.1] | 76.3 [8.0] | 72.1 [11.0] | .24^b^ |  | 73.2 [8.1] | 75.6 [9.9] | 72.9 [10.2] | .58^b^ |
| **SBP right (mmHg)^2^** | 133.4 [14.8] | 133.0 [15.9] | 133.3 822.4] | .99^b^ |  | 135.2 [15.6] | 130.0 [17.1] | 131.2 [21.2] | .54^b^ |
| **Total cholesterol (mmol/L)^2^** | 5.7 [1.1] | 5.3 [0.9] | 5.5 [1.1] | .34^a^ |  | 6.0 [1.2] | 5.2 [0.7] | 5.5 [1.2] | .02^a^* |
| **HDL (mmol/L)^2^** | 1.9 [0.6] | 1.7 [0.3] | 1.9 [0.5] | .42^a^ |  | 2.0 [0.6] | 1.7 [0.5] | 1.9 [0.6] | .31^a^ |
| **LDL (mmol/L)^2^** | 3.5 [1.0] | 3.3 [0.6] | 3.2 [0.9] | .56^a^ |  | 3.8 [1.0] | 3.1 [0.6] | 3.3 [1.0] | .06^a^ |
| **Glucose (mmol/L)^2^** | 5.5 [0.7] | 5.3 [0.7] | 5.5 [0.5] | .39^a^ |  | 5.5 [0.5] | 5.4 [0.7] | 5.5 [0.7] | .44^a^ |
| **HbA1c (%)^2^** | 6.5 [0.3] | 5.5 [0.4] | 5.5 [0.4] | .37^a^ |  | 5.4 [0.2] | 5.5 [0.6] | 5.5 [0.5] | .92^a^ |
| **hsCRP (mg/L)^2^** | 2.2 [3.4] | 2.9 [5.2] | 1.8 [1.8] | .39^a^ |  | 3.4 [9.8] | 1.9 [2.4] | 2.6 [4.7] | .57^a^ |
| **TG (mmol/L)^2^** | 0.9 [0.4] | 0.9 [0.3] | 0.9 [0.4] | .78^a^ |  | 0.9 [0.5] | 1.0 [0.5] | 1.0 [0.4] | .64^a^ |
| **HADS total score^2^** | 7.1 [4.5] | 6.6 [4.7] | 4.9 [3.4] | .13^a^ |  | 6.6 [4.5] | 6.1 [4.7] | 5.0 [3.6] | .39^a^ |
| **Sleep problem index^2^** | 5.2 [1.4] | 5.2 [1.9] | 4.7 [1.2] | .54^a^ |  | 5.3 [1.5] | 5.4 [1.6] | 4.8 [1.3] | .37^a^ |
| **Cardiorespiratory fitness testing** | | | | | | | | | |
| **CRF (****mL·kg^-1^·min^-1^)^2^** | 31.9 [7.2] | 31.9 [5.4] | 32.3 [6.5] | .96^b^ |  | 31.3 [8.0] | 29.7 [6.2] | 31.8 [7.3] | .64^b^ |
| **VO_2max_ or VO_2peak_^e, 1^** | 24/19 | 9/13 | 18/15 | .49^d^ |  | 22/18 | 8/10 | 19/11 | .26^d^ |
| **RER^2^** | 1.07 [0.08] | 1.04 [0.08] | 1.10 [0.09] | .10^a^ |  | 1.08 [0.09] | 1.04 [0.08] | 1.09 [0.09] | .14^a^ |
| **Maximal exercise intensity (6-20 Borg scale)^2^** | 17.3 [1.7] | 17.5 [1.5] | 17.4 [1.8] | .92^a^ |  | 17.2 [1.5] | 17.4 [1.5] | 17.4 [1.4] | .85^a^ |
| **HR_peak_ (beats/min)^2^** | 160.7 [11.7] | 155.7 [12.3] | 158.5 [12.4] | .44^a^ |  | 155.2 [15.6] | 152.0 [17.0] | 155.0 [12.7] | .88^a^ |

^1^ – results represent number of participants; ^2^ – results represent mean [±standard deviation]; *p < 0.050; ^a^ - Kruskal – Wallis test, ^b^ - ANOVA, ^c^ - ln transformed for the analysis, raw data reported; ^d^ - Pearson Chi-Square, ^e^– Number of CRF measurements on a treadmill, where a plateau in oxygen uptake (i.e., VO_2max_) and RER ≥1.05 were observed; and those who did not meet the aforementioned criteria (and were therefore referred to as VO_2peak_); M: men; W: women; P: primary education; S: secondary education; T: tertiary education; Y: yes; N: no; BMI: body mass index; RHR: resting heart rate; DBP: diastolic blood pressure; SBP: systolic blood pressure; HDL: high-density lipoprotein; LDL: low-density lipoprotein; HbA1c: glycated hemoglobin; hsCRP: high sensitivity C-reactive protein; TG: triglycerides; HADS: Hospital Anxiety and Depression Scale; CRF: cardiorespiratory fitness measured as VO_2peak_ or VO_2max_; VO_2peak_: peak oxygen uptake; VO_2max_: maximal oxygen uptake; RER: maximal respiratory exchange ratio; HR_peak_: peak heart rate.
