# Supplementary material for: 5 Years of Exercise Intervention Did Not Benefit Cognition Compared to the Physical Activity Guidelines in Older Adults, but Higher Cardiorespiratory Fitness Did. A Generation 100 Substudy
Source: Front Aging Neurosci. 2021 Nov 16;13:742587. doi: 10.3389/fnagi.2021.742587 (PMC8637860; doi:10.3389/fnagi.2021.742587)
Supplement: Supplementary file 2 [file Table_2.docx]

*Supplementary Table 2.* ***Results of the linear mixed models assessing time and group*time interaction effects on cardiorespiratory fitness (CRF) in the Moderate Intensity Continuous training group (MICT) and the High Intensity Interval Training group (HIIT) compared to the control group. The table includes the analysis of all study participants who achieved VO_2peak_ as well as the analysis including only participants who achieved VO_2max_ on a treadmill, with RER≥1.05. The first model also assesses if the two CRF measurement types (VO_2peak_ vs VO_2max_) differentiated its value.***

|  | **All participants** | **VO_2max_ participants** |
| --- | --- | --- |
| **Predictors** | Coef. [CI] | Coef. [CI] |
| **1 year** | 1.36* [0.13,2.59] | 0.86 [-1.03,2.74] |
| **3 years** | 0.07 [-1.26,1.41] | -0.58 [-2.51,1.35] |
| **5 years** | -1.23 [-2.60,0.14] | -1.51 [-3.49,0.47] |
| **MICT*1 year** | 0.99 [-1.15,3.12] | -0.89 [-4.60,2.81] |
| **MICT*3 years** | -0.34 [-2.61,1.93] | -1.69 [-5.41,2.02] |
| **MICT*5 years** | 0.24 [-2.04,2.52] | -0.27 [-4.29,3.75] |
| **HIIT*1 year** | 0.20 [-1.66,2.06] | 0.35 [-2.37,3.07] |
| **HIIT*3 years** | 1.00 [-0.96,2.97] | 0.79 [-1.93,3.51] |
| **HIIT*5 years** | 0.96 [-1.11,3.03] | 1.24 [-1.78,4.26] |
| **VO_2peak_** | -0.76 [-1.63,0.11] | - |
| **N** | 343 | 201 |

*p < 0.050; Coef.: coefficients; CI: confidence intervals; 1 year: one-year follow-up; 3 years: three-year follow-up; 5 years: five-year follow-up; N: number of observations.
Besides the variables shown in the table, the model controlled for age at inclusion, sex, and education.
