# Supplementary material for: 5 Years of Exercise Intervention Did Not Benefit Cognition Compared to the Physical Activity Guidelines in Older Adults, but Higher Cardiorespiratory Fitness Did. A Generation 100 Substudy
Source: Front Aging Neurosci. 2021 Nov 16;13:742587. doi: 10.3389/fnagi.2021.742587 (PMC8637860; doi:10.3389/fnagi.2021.742587)
Supplement: Supplementary file 4 [file Table_4.docx]

*Supplementary Table 4. Results of the linear mixed model (Supplementary Model 2). The model assessed group*time interaction, change in cardiorespiratory fitness (CRF), and time effects on cognitive test performance during the intervention in the High Intensity Interval Training group (HIIT) and Moderate Intensity Continuous Training group (MICT) compared to the control group, like Model 2, but only included participants who achieved VO_2max_ on a treadmill, with RER≥1.05.*

|  | **Spatial memory** | **Verbal memory** | **Pattern separation** | **Processing speed** | **Working memory** | **Planning ability** |
| --- | --- | --- | --- | --- | --- | --- |
| **Predictors** | Coef. [CI] | Coef. [CI] | Coef. [CI] | Coef. [CI] | Coef. [CI] | Coef. [CI] |
| **CRF change** | 0.01 [-0.02,0.04] | 0.01 [-0.02,0.04] | -0.04* [-0.07,-0.01] | 0.01 [-0.02,0.04] | 0.02 [-0.01,0.05] | -0.00 [-0.04,0.03] |
| **1 year** | 0.44* [0.07,0.81] | -0.13 [-0.45,0.20] | 0.14 [-0.22,0.50] | 0.41* [0.08,0.73] | 0.17 [-0.16,0.51] | 0.49 [-0.00,0.97] |
| **3 years** | 0.15 [-0.23,0.54] | 0.12 [-0.21,0.45] | 0.43* [0.06,0.81] | 0.38* [0.05,0.72] | -0.07 [-0.42,0.27] | 0.93***# [0.42,1.43] |
| **5 years** | 0.17 [-0.23,0.58] | -0.33 [-0.69,0.02] | -0.01 [-0.39,0.38] | 0.33 [-0.02,0.68] | -0.20 [-0.56,0.15] | 0.65* [0.09,1.21] |
| **MICT*1 year** | 0.06 [-0.61,0.74] | -0.03 [-0.67,0.61] | -0.28 [-0.94,0.38] | 0.17 [-0.45,0.80] | -0.01 [-0.66,0.65] | 0.67 [-0.11,1.45] |
| **MICT*3 years** | 0.15 [-0.52,0.83] | 0.06 [-0.57,0.69] | -0.72* [-1.38,-0.06] | -0.06 [-0.68,0.56] | 0.25 [-0.40,0.89] | -0.03 [-0.81,0.75] |
| **MICT*5 years** | 0.41 [-0.35,1.17] | 0.05 [-0.63,0.72] | 0.34 [-0.40,1.08] | 0.41 [-0.28,1.10] | 0.52 [-0.20,1.24] | -0.08 [-0.98,0.82] |
| **HIIT*1 year** | 0.29 [-0.21,0.79] | 0.07 [-0.39,0.53] | 0.17 [-0.32,0.66] | -0.25 [-0.71,0.21] | -0.12 [-0.59,0.36] | -0.09 [-0.67,0.49] |
| **HIIT*3 years** | 0.08 [-0.44,0.60] | -0.05 [-0.52,0.42] | -0.10 [-0.60,0.40] | -0.04 [-0.51,0.42] | 0.06 [-0.42,0.55] | -0.55 [-1.16,0.06] |
| **HIIT*5 years** | 0.30 [-0.29,0.89] | 0.26 [-0.26,0.79] | 0.26 [-0.32,0.83] | -0.19 [-0.73,0.35] | 0.38 [-0.16,0.93] | -0.22 [-0.96,0.51] |
| **N** | 195 | 181 | 194 | 194 | 194 | 165 |

*p < 0.050; ***p ≤ 0.001; #: effect still significant after Holm-Bonferroni correction; Coef.: coefficients; CI: confidence intervals; CRF, cardiorespiratory fitness, change: change in VO_2peak_ between each time point; 1 year: one-year follow-up; 3 years: three-year follow-up; 5 years: five-year follow-up; N: number of observations.
Besides the variables shown in the table, the model controlled for age at inclusion, sex, and education.
