# Supplementary material for: 5 Years of Exercise Intervention Did Not Benefit Cognition Compared to the Physical Activity Guidelines in Older Adults, but Higher Cardiorespiratory Fitness Did. A Generation 100 Substudy
Source: Front Aging Neurosci. 2021 Nov 16;13:742587. doi: 10.3389/fnagi.2021.742587 (PMC8637860; doi:10.3389/fnagi.2021.742587)
Supplement: Supplementary file 5 [file Table_5.docx]

*Supplementary Table 5. Results of the linear regression (Model 3). The model assessed if CRF at baseline could predict cognitive performance after 1, 3 and 5 years of intervention, like Model 3, but included only participants who achieved VO_2max_ on a treadmill, with RER≥1.05. A separate regression analysis was run for each time point.*

|  | **Spatial memory** | **Verbal memory** | **Pattern separation** | **Processing speed** | **Working memory** | **Planning ability** |
| --- | --- | --- | --- | --- | --- | --- |
| **Time point** | Coef. [CI] | Coef. [CI] | Coef. [CI] | Coef. [CI] | Coef. [CI] | Coef. [CI] |
| **1 year** | 0.03 [-0.02,0.07] | 0.01 [-0.04,0.06] | 0.01 [-0.04,0.05] | 0.06* [0.01,0.10] | 0.00 [-0.05,0.05] | 0.04 [-0.01,0.09] |
| **3 years** | 0.02 [-0.02,0.07] | 0.04* [0.01,0.08] | 0.01 [-0.03,0.05] | 0.06** [0.02,0.10] | 0.02 [-0.03,0.06] | 0.03 [-0.01,0.08] |
| **5 years** | 0.02 [-0.02,0.06] | 0.04* [0.00,0.09] | 0.00 [-0.04,0.05] | 0.04 [-0.01,0.09] | 0.01 [-0.05,0.06] | 0.02 [-0.04,0.09] |

*p < 0.050; **p ≤ 0.010; Coef.: coefficients; 95% CI: confidence intervals; CRF: cardiorespiratory fitness; 1 year: one-year follow-up; 3 years: three-year follow-up; 5 years: five-year follow-up.
Besides the variables shown in the table, the model controlled for age at the time of testing, sex, and education. No tests were significant after Holm-Bonferroni correction.
